# Effects of different additives on fermentation quality, mycotoxin concentrations, and microbial communities in high-moisture corn kernels during wet storage

**DOI:** 10.3389/fmicb.2024.1508842

**Published:** 2024-12-04

**Authors:** Xingya Wang, Linlin Sun, Shoumei Zhang, Yuqiu Guo, Lirong Chen, Kuijie Gong, Kaichang Liu

**Affiliations:** ^1^Crop Research Institute, Shandong Academy of Agricultural Sciences, Jinan, China; ^2^Shandong Academy of Agricultural Sciences, Jinan, China

**Keywords:** high-moisture corn kernels, wet storage, microbial community, lactic acid bacteria, quality safety

## Abstract

**Introduction:**

The moisture content of corn kernels at harvest in China is relatively high, and wet storage effectively preserves high-moisture corn kernels. However, ensuring effective fermentation during storage is crucial.

**Methods:**

To address this, we systematically investigated the variations in fermentation quality, mycotoxin concentrations, and microbial community composition under different additive treatments. The treatments included CK (control, deionized water), LAB homo- and hetero-lactic acid bacteria: *Lactiplantibacillus plantarum* and *Weissella confusa* MF01, and EN (cellulase), followed by 60 and 90 days of fermentation.

**Results:**

The results indicated that both LAB and EN treatments significantly reduced the concentrations of deoxynivalenol (DON), aflatoxin B_1_ (AFB_1_), and zearalenone (ZEN) compared to CK during the wet storage of high-moisture corn kernels. LAB treatment notably increased lactic and acetic acid levels, decreased pH and NH_3_-N content, and improved crude protein (CP, 8.24% DM) and starch content (73.01% DM) compared to CK. LAB treatment also reduced water-soluble carbohydrate (WSC) content (5.05% DM). Microbial diversity was reduced in the LAB treatment, as evidenced by decreases in both common and unique operational taxonomic units, while the relative abundance of *Weissella* increased after 60 days compared to CK. In contrast, despite higher lactic and acetic acid levels in the EN treatment, the pH did not decrease significantly due to higher NH_3_-N content. Overall, the LAB treatment outperformed other treatments by achieving lower mycotoxin concentrations, better fermentation quality, and superior preservation of nutritional components.

**Discussion:**

This study provides valuable theoretical support and practical guidance for improving the wet storage of high-moisture corn kernels and enhancing their safety and nutritional value during storage.

## Introduction

The moisture content of corn kernels at harvest in China is relatively high, often exceeding 30% (Wang et al., 2019). Direct mechanized harvesting leads to a higher rate of kernel breakage, making the embryo and seed coat more susceptible and providing nutrients for mold growth (Maiorano et al., 2014). High-moisture corn with a high breakage rate faces many issues, including spoilage, toxin production, and nutrient degradation (Neme and Mohammed, 2017; Channaiah and Maier, 2014). Additionally, to reduce moisture content, post-harvest high-moisture corn requires costly procedures such as drying and aeration (Wang et al., 2022; Bullerman and Bianchini, 2014). These factors limit the development of mechanized grain harvesting for corn. Therefore, it is crucial to explore simple, economical, and effective storage methods. Studies have shown that wet storage of high-moisture corn kernels can preserve corn better, prevent mold growth, maintain nutritional content, and reduce waste, representing a novel approach for utilizing high-moisture corn kernels (da Silva et al., 2015; de Almeida Carvalho-Estrada et al., 2020).

Previous studies have indicated that the timing of crop harvesting significantly influences the species, quantity, and toxin content of fungi (Torres et al., 2014). Delaying harvesting to later growth stages increases the risk of fungal infection, growth, and toxin accumulation (Kaaya et al., 2005). Moreover, fungi and toxins present in crops prior to harvest have a substantial impact on subsequent fermentation characteristics and quality (Neme and Mohammed, 2017; Mansfield and Kuldau, 2007). However, previous research on corn toxins has often treated these processes separately, focusing exclusively on either the crop growth period or wet storage process (Richard et al., 2007; Dell’Orto et al., 2015), without an integrated and cohesive investigation across both harvesting and storage stages. Conducting a systematic study of fungal infection and toxin content in corn kernels during high-moisture harvest, along with the changes in fungal toxins after wet storage, is of great importance.

Using appropriate wet storage additives can enhance lactic acid bacteria fermentation, inhibit spoilage bacteria growth, and improve nutritional value during wet storage (Der Bedrosian and Kung, 2019; Queiroz et al., 2013). Inoculating with lactic acid bacteria before ensiling is a common practice for improving fermentation quality and enhancing resistance to oxygen exposure (Muck et al., 2018). Current research has shown that the simultaneous addition of homo- and hetero-lactic acid bacteria leads to better fermentation results (Muck et al., 2018). However, studies on other combinations of lactic acid bacteria are limited, with most research focusing on combinations of *Lactiplantibacillus plantarum* and *Lactiplantibacillus buchneri* (Weinberg et al., 2002; Adesogan and Salawu, 2004). As an enzyme additive, cellulase can aid in breaking down plant cell walls during various stages of ensiling, thereby enhancing fermentation and potentially reducing the volatility of fatty acid production (Lynch et al., 2014; Ruiz et al., 2013). However, research on cellulase application in the wet storage of high-moisture corn kernels remains limited and requires comprehensive exploration. Overall, previous studies have shown the effectiveness of certain additives in improving the fermentation process and enhancing fermentation quality (Kharazian et al., 2017; Yuan et al., 2015). However, there is a lack of systematic research on the effects of different additive combinations in the wet storage of high-moisture corn kernels, particularly regarding their impact on microbial community diversity and abundance.

In this study, ZD958 corn kernels with a moisture content of 32% were harvested and subjected to wet storage. Three treatments were set: the addition of deionized water (CK), the addition of homo- and hetero-lactic acid bacteria (*Lactiplantibacillus plantarum* and *Weissella confusa* MF01, LAB), and the addition of cellulase (EN). The study systematically investigated changes in mycotoxin concentrations throughout both the harvesting and wet storage processes. It explored the effects of different additive treatments on the chemical composition and fermentation characteristics of high-moisture corn kernels during wet storage. Furthermore, using advanced next-generation high-throughput sequencing methods, we aimed to study the dynamics of microbial community structures under different additive treatments. The purpose of this study was to provide a certain theoretical basis and technical support for the utilization of high-moisture corn kernels.

## Materials and methods

### Corn harvest and wet storage treatment

Corn was planted for wet storage in 2022 in the Zhangqiu Experimental Station of the Shandong Academy of Agricultural Sciences (Shandong Province, China; 117.48°E, 36.78°N). The maize hybrid used was Zhengdan 958 (ZD958), planted on June 20, 2022, and harvested on October 20, 2022. Fresh corn kernels were dried at 75°C in a forced-draft oven to constant weight and then weighed to calculate the grain moisture content, which was 32%. The chemical composition, fermentation parameters, and mycotoxin concentrations of fresh, high-moisture corn kernels before fermentation are listed in Table 1. The fresh, high-moisture corn kernels were crushed to 0.5 cm (with intact kernels comprising no more than 5% and the fine powder no more than 20%) using a stainless-steel grinder (DX-55, Daxiang, China) and subsequently fermented in anaerobic fermentation bags (23 cm × 40 cm). The crushed corn kernels were divided into three treatments: the addition of deionized water (CK), the addition of homo- and hetero-lactic acid bacteria (LAB), and the addition of cellulase (EN). The LAB treatment additive was a mix of lactic acid bacteria consisting of a combination of *Lactiplantibacillus plantarum* (Zhenjiang Tianyi Biotechnology, China) and *Weissella confusa* MF01 at a ratio of 1:1, which was inoculated at a concentration of 10^5^ CFU/g of fresh material. *Weissella confusa* MF01 was isolated from wheat bran by the Crop Research Institute of Shandong Academy of Agricultural Sciences and was preserved at the China General Microbiological Culture Collection Center (Preservation Number: M20221635). The EN activity was 10,000 U/g (Nanjing Pangbo Biotechnology, China), with an additional amount of 200 U/g. The LAB and EN additives were prepared by mixing the additives with 10 mL of sterile distilled water and spraying them onto 750 g of crushed corn kernels; only 10 mL of sterile distilled water was added in the CK treatment. The mixed materials were placed into anaerobic fermentation bags, and air was eliminated by a vacuum sealer. Three replicates for each treatment were made and fermented for 60 and 90 days. In total, 18 bags (3 replicates × 3 treatments × 2 fermentation time) of high-moisture corn kernels were conserved at room temperature.

**Table 1:** Chemical composition, fermentation parameters, and mycotoxin concentrations of fresh, high-moisture corn kernels.

Items	Fresh, high-moisture corn kernels
DM (% FM)	68.14 ± 0.34
Ash (% DM)	1.25 ± 0.01
CP (% DM)	8.00 ± 0.08
EE (% DM)	3.90 ± 0.02
Starch (% DM)	70.95 ± 1.00
WSC (% DM)	10.53 ± 0.06
NDF (% DM)	8.70 ± 0.12
ADF (% DM)	3.90 ± 0.08
pH	6.67 ± 0.03
Lactic acid (% DM)	0.23 ± 0.03
Acetic acid (% DM)	0.20 ± 0.02
NH_3_-N (% DM)	0.13 ± 0.02
DON (μg·kg^−1^ DM)	0.68 ± 0.11
AFB_1_ (μg·kg^−1^ DM)	0.53 ± 0.04
ZEN (μg·kg^−1^ DM)	14.25 ± 0.74

DM, Dry matter; FM, Fresh matter; Ash, Crude ash; CP, Crude protein; EE, Ether extract; WSC, Water-soluble carbohydrate; NDF, Neutral detergent fiber; ADF, Acid detergent fiber.

### Analysis of mycotoxin concentrations, chemical composition, and fermentation characteristics

The fresh and fermented corn kernels were dried to a constant weight at 70°C to measure the dry matter content. Dried samples were ground using a stainless-steel grinder and sieved through a 40-mesh screen to analyze the mycotoxin concentrations and other chemical compositions. An enzyme-linked immunosorbent assay (ELISA) was used to determine mycotoxin concentrations. Crude ash (Ash) was measured according to the standard procedures of the Association of Official Analytical Chemists. Crude protein (CP) content was determined using the Kjeldahl method for nitrogen estimation. The ether extract (EE) was determined using the Soxhlet extraction method. Starch and water-soluble carbohydrates (WSC) were quantified using the anthrone colorimetric method. Neutral detergent fiber (NDF) and acid detergent fiber (ADF) were analyzed following the procedures outlined by Van Soest et al. (1991).

Approximately 20 g of silage sample and 180 mL of sterilized water were blended and then passed through a 0.22 μm membrane to analyze fermentation characteristics. pH value was measured using a pH meter (FE28-bio, METTLER TOLEDO). Organic acid content was determined using a high-performance liquid chromatography system (Agilent 1100, Agilent) equipped with a C18 column (4.6 mm × 150 mm, 5 μm) with a mobile phase of 0.05 mol/L phosphate buffer at pH 2.8 and methanol, injecting 10 μL, detection wavelength at 210 nm, column temperature at 40°C, and a flow rate of 1.0 mL/min. Ammoniacal nitrogen (NH_3_-N) content was measured using the phenol-hypochlorite colorimetric method.

### Microbiome analysis

DNA was extracted from corn kernel samples using the CTAB extraction method, and its purity was assessed through 1% agarose gel electrophoresis. The extracted DNA was then diluted to 1 ng/μL with sterile water. This diluted genomic DNA serves as the template for PCR amplification of the bacterial 16S rRNA V4 region. All PCR reactions were carried out with 15 μL of Phusion® High-Fidelity PCR Master Mix (New England Biolabs), 0.2 μM of forward and reverse primers (341F: CCTAYGGGRBGCASCAG; 806R: GGACTACNNGGGTATCTAAT), and approximately 10 ng template DNA. Thermal cycling consisted of initial denaturation at 98°C for 1 min, followed by 30 cycles of denaturation at 98°C for 10 s, annealing at 50°C for 30 s, and elongation at 72°C for 30 s and 72°C for 5 min. We then mixed the same volume of 1X loading buffer (containing SYB green) with PCR products and operated electrophoresis on 2% agarose gel for detection. PCR products were mixed in equidensity ratios. Then, the mixture of PCR products was purified using a universal DNA purification kit (TianGen, China, Catalog #: DP214). Sequencing libraries were generated using the NEB Next® Ultra™ II FS DNA PCR-free Library Prep Kit (New England Biolabs, United States, Catalog #: E7430L) following the manufacturer’s recommendations, and indexes were added. The library was checked with Qubit, real-time PCR was used for quantification, and a bioanalyzer was used for size distribution detection. Quantified libraries were pooled and sequenced on Illumina platforms according to the effective library concentration and data amount required.

Based on the barcode and PCR amplification primer sequences, data for each sample were extracted from the sequencing data. After removing the barcode and primer sequences, reads were assembled for each sample using FLASH (V1.2.7, http://ccb.jhu.edu/software/FLASH/), generating concatenated sequences referred to as raw tags. These raw tags were subjected to stringent filtering to produce high-quality tags, referred to as clean tags. The final effective tags were obtained through the quality control process outlined in QIIME (V1.9.1, http://qiime.org/scripts/split_libraries_fastq.html).

Subsequently, Uparse software was used to cluster the effective tags from all samples, defaulting to clustering sequences into Operational Taxonomic Units (OTUs) at 97% sequence identity. OTU representative sequences were taxonomically annotated using the Mothur method, comparing them against a reference database while simultaneously calculating the relative abundance of each OTU across samples. Data analysis was performed using QIIME.

### Statistical analysis

The data were analyzed using the SPSS software (Version 19.0: IBM Corp., Armonk, NY). The mycotoxin concentrations, chemical composition, and fermentation characteristics were analyzed using a two-way ANOVA with Duncan’s multiple-range test. Differences were judged with the LSD test using a 0.05 level of significance. The QIIME software was used for microbial community data analysis.

## Results

### Characteristics of fresh, high-moisture corn kernels

For freshly harvested high-moisture corn kernels, the DM content was 68.14% of the original weight (Table 1). The chemical components, including Ash, CP, EE, Starch, WSC, NDF, and ADF, were 1.25, 8.00, 3.90, 70.95, 10.53, 8.70, and 3.90% of DM, respectively. The pH values of lactic acid, acetic acid, and NH_3_-N contents were 6.67, 0.23, 0.20, and 0.13% of DM, respectively. Moreover, the concentrations of DON, AFB_1_, and ZEN were 0.68, 0.53, and 14.25 μg·kg^−1^, respectively.

### The concentrations of DON, AFB_1_, and ZEN in wet-stored high-moisture corn kernels

**Figure 1:**
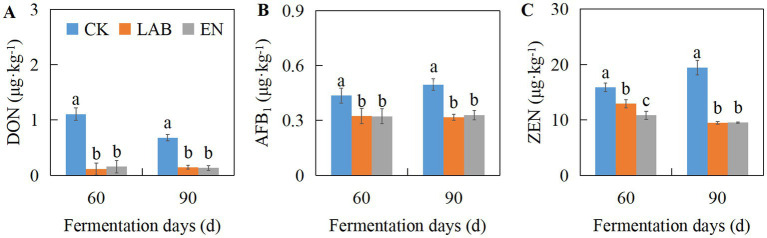
The concentrations of DON **(A)**, AFB_1_ **(B)**, and ZEN **(C)** in high-moisture corn kernels after 60 and 90 days of wet storage. CK, The addition of deionized water; LAB, The addition of homo- and hetero-lactic acid bacteria; EN, The addition of cellulase.

As shown in Figure 1 and Supplementary Table S1, the additive treatments significantly affected the concentrations of DON, AFB_1_, and ZEN (*p* < 0.05). On days 60 and 90, compared with the CK treatment, the LAB and EN treatments significantly decreased the concentrations of DON, AFB_1_, and ZEN (*p* < 0.05). The concentrations of DON, AFB_1_, and ZEN in the LAB and EN treatments decreased by 84.3, 30.8, and 35.1%, and 83.2, 29.8, and 41.4%, respectively. Overall, after fermentation, the concentrations of DON, AFB_1_, and ZEN in all treatments ranged from 0.1–1.1, 0.3–0.5, and 9.5–19.5 μg·kg^−1^, respectively, remaining relatively low.

### Chemical composition of wet-stored high-moisture corn kernels

There were no significant differences in DM and EE contents between fermentation days or additive treatments (Table 2). Fermentation days and additive treatments had significant differences in starch, WSC, and NDF contents (*p* < 0.05). During the fermentation, starch, and WSC contents of the three groups gradually decreased, and on days 60 and 90 of fermentation, the LAB treatment significantly increased starch content compared to CK and EN treatments (*p* < 0.05), with increases of 5.7 and 2.8%, respectively. On days 60 and 90, the LAB and EN treatments significantly decreased WSC content compared to CK treatment (*p* < 0.05), with decreases of 29.0 and 29.6%, respectively. Fermentation days had no significant effect on ash, CP, and ADF contents, but there were significant differences between the additive treatments (*p* < 0.05). On days 60 and 90, the LAB treatment significantly increased CP content and decreased ash content compared to CK and EN treatments (*p* < 0.05). The CP content increased by 1.5 and 5.3%, respectively, while the ash content decreased by 21.8 and 66.5%, respectively. The ADF content in the LAB and EN treatments was significantly lower than in the CK treatment (*p* < 0.05).

**Table 2:** Chemical composition of high-moisture corn kernels after 60 and 90 days of wet storage.

Items		DM (%FM)	Ash (%DM)	CP (%DM)	EE (%DM)	Starch (%DM)	WSC (%DM)	NDF (%DM)	ADF (%DM)
60 days	CK	65.12aA	1.38bA	8.13bA	3.70aB	72.23bA	9.75aA	8.22bB	3.30aA
	LAB	65.10aA	0.99cB	8.24aA	4.20aA	73.60aA	6.38bA	8.70aA	2.50bA
	EN	66.53aA	3.09aB	7.77cA	4.30aA	71.61bA	5.49bA	8.43bA	2.40bA
90 days	CK	66.00aA	1.30bB	8.10bA	4.30aA	66.10cB	4.86aB	8.67aA	3.60aA
	LAB	64.87aA	1.10cA	8.24aA	4.30aA	72.43aB	3.72bB	8.88aA	2.80bA
	EN	64.65aA	3.14aA	7.88cA	4.00aA	70.41bB	4.10bB	8.34bA	1.70cB
SEM		0.252	0.275	0.044	0.086	0.600	0.498	0.061	0.158
*p*	T	0.581	<0.001	<0.001	0.461	<0.001	<0.001	<0.001	<0.001
	F	0.408	0.34	0.219	0.421	<0.001	<0.001	0.010	0.778
	T × F	0.161	0.089	0.059	0.111	<0.001	<0.001	0.010	0.005

DM, Dry matter; FM, Fresh matter; Ash, Crude ash; CP, Crude protein; EE, Ether extract; WSC, Water-soluble carbohydrate; NDF, Neutral detergent fiber; ADF, Acid detergent fiber; CK, The addition of deionized water; LAB, The addition of homo- and hetero-lactic acid bacteria; EN, The addition of cellulase; SEM, Standard error mean; T, Treatment; F, Fermentation day; T × F, Interactive effects of treatments and fermentation days. Capital letters indicate significance within fermentation days at *p* < 0.05, and small letters indicate significance within additives at a *p* value of < 0.05.

### Fermentation characteristics of wet-stored high-moisture corn kernels

Fermentation days and additive treatments significantly differed in pH value, lactic acid, acetic acid, and NH_3_-N contents (Table 3, *p* < 0.05). During fermentation, the lactic acid, acetic acid, and NH_3_-N contents of the three treatments gradually increased. On days 60 and 90, compared with CK and EN treatments, the LAB treatment significantly decreased both pH value and NH_3_-N content (*p* < 0.05). The pH decreased by 11.3 and 17.7%, respectively, and NH_3_-N content decreased by 15.3 and 25.6%, respectively. The lactic acid and acetic acid contents were similar between the LAB and EN treatments and were higher than in the CK treatment (*p* < 0.05). Interestingly, despite the high levels of lactic acid and acetic acid in the EN treatment (*p* < 0.05), the pH value was significantly higher than in the CK treatment (*p* < 0.05, 5.1 vs. 4.7).

**Table 3:** Fermentation characteristics of high-moisture corn kernels after 60 and 90 days of wet storage.

Items		pH	Lactic acid (% DM)	Acetic acid (% DM)	NH_3_-N (% DM)
60 days	CK	4.77bA	1.33bB	0.53bB	2.73bB
	LAB	4.20cA	2.41aB	0.60aB	2.00cB
	EN	5.29aA	2.43aB	0.65aB	3.24aB
90 days	CK	4.69bA	1.97bA	0.69bA	3.30bA
	LAB	4.19cA	2.87aA	0.81aA	3.17cA
	EN	4.92aA	2.80aA	1.00aA	3.64aA
SEN		0.099	0.129	0.041	0.128
*p*	T	<0.001	<0.001	0.004	<0.001
	F	0.016	<0.001	<0.001	<0.001
	T × F	0.057	0.147	0.208	<0.001

CK, Addition of deionized water; LAB, Addition of homo- and hetero-lactic acid bacteria; EN, Addition of cellulase; SEM, Standard error mean; T, Treatment; F, Fermentation day; T × F, Interactive effects of treatments and fermentation days. Capital letters indicate significance within fermentation days at *p* < 0.05, and small letters indicate significance within additives at a *p* value of < 0.05.

### Microbial community in fresh and wet storage high-moisture corn kernels

In total, 1,116,312 effective 16S rRNA sequences were obtained from 12 samples and clustered into 320 OTUs (Table 4). Alpha diversity analysis was used to evaluate species richness and diversity within individual samples. The Good’s coverage index assessed sequencing depth adequacy, with values close to 1 (or 100%) indicating adequate coverage of species in the sample. Observed features represented the number of OTUs detected, while the Chao1 index estimated the total potential number of species in the community, with higher values indicating greater species potential.

**Table 4:** Differences in bacterial community diversity and richness between fresh corn kernels and corn kernels after 60 days of wet storage.

Taxonomy	Observed features	Chao1	Dominance	Shannon	Simpson	Goods coverage
BF	93	99	0.495	1.133	0.505	1
CK	162	184	0.127	3.802	0.874	1
LAB	134	145	0.374	2.408	0.626	1
EN	136	136	0.364	2.628	0.636	1

BF, Fresh corn kernels; CK, The addition of deionized water; LAB, The addition of homo- and hetero-lactic acid bacteria; EN, The addition of cellulase.

The Dominance index measures the presence of dominant species or the significant predominance of one or more species within the community; higher values indicate a more pronounced presence of dominant species and reduced biodiversity. The Shannon index represents species diversity in the sample, with higher values indicating greater community diversity. The Simpson index primarily reflects the evenness and diversity of species in the community; values closer to 0 indicate higher diversity and evenness, while values closer to 1 indicate a greater dominance of certain species and reduced evenness.

In this study, the Good’s coverage was approximately 1 across all treatments. Chao1, Observed features, the Shannon index, and the Simpson index increased across all treatments after fermentation, while the Dominance index decreased. Among the fermentation treatments, the LAB and EN groups exhibited lower Observed features, Chao1, Shannon, and the Simpson index compared to CK but showed higher dominance.

**Figure 2:**
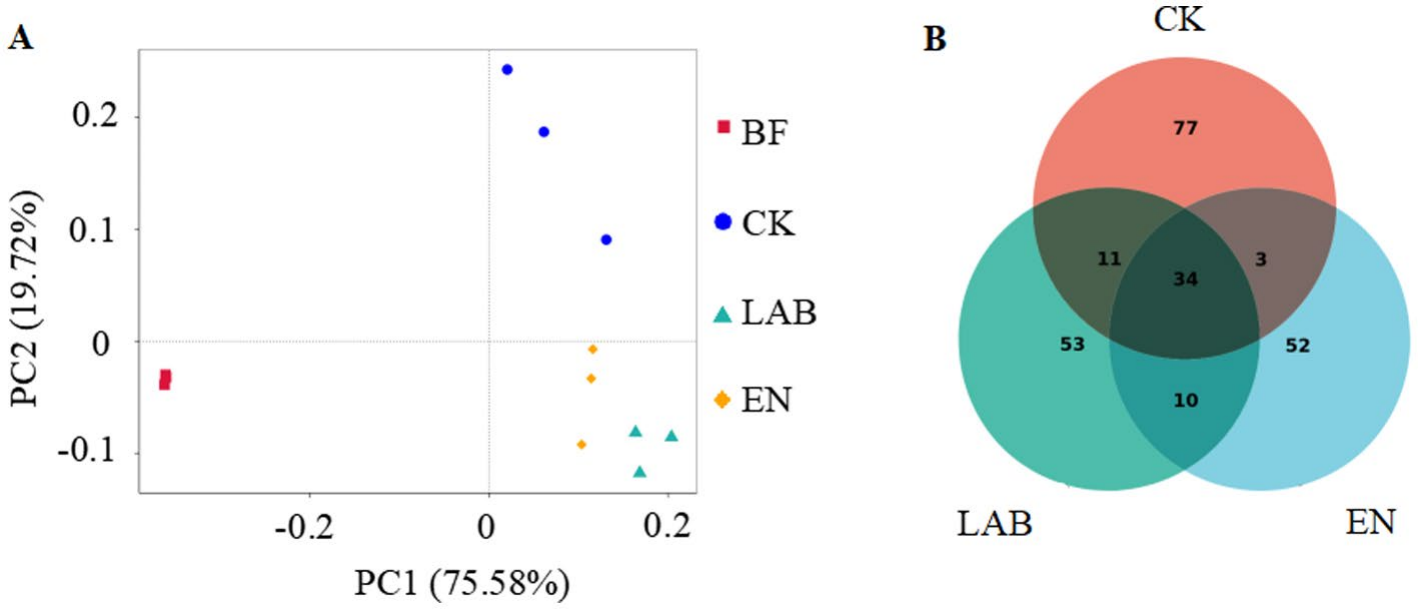
**(A)** Principle coordinate analysis (PCoA) of the bacterial community in fresh and wet storage samples based on Bray-Curtis distance. **(B)** Venn diagram depicting unique or common bacterial OTUs in wet storage samples after 60 days of wet storage. BF, Fresh corn kernels; CK, The addition of deionized water; LAB, The addition of homo- and hetero-lactic acid bacteria; and EN, The addition of cellulase.

Principle coordinate analysis extracts the main elements and structures from multidimensional data using eigenvalues and eigenvectors. The principal coordinate combination with the highest contribution rate is selected for graphical display. If the distance between samples is closer, it indicates greater similarity in species composition, meaning that samples with similar community structures tend to cluster together, while samples with significant community differences will be farther apart. PC1 and PC2 explained 75.6 and 19.7% of the total variation, respectively (Figure 2A). There were significant differences between fermented and freshly harvested. The fermentation treatments showed a separate cluster from each other, indicating significant differences in microbial structure.

The LAB and EN treatments decreased the number of OUTs compared with fresh, high-moisture corn kernels (Figure 2B). Among all fermentation treatments, the LAB and EN treatments had the lowest total number and unique OUTs.

**Figure 3:**
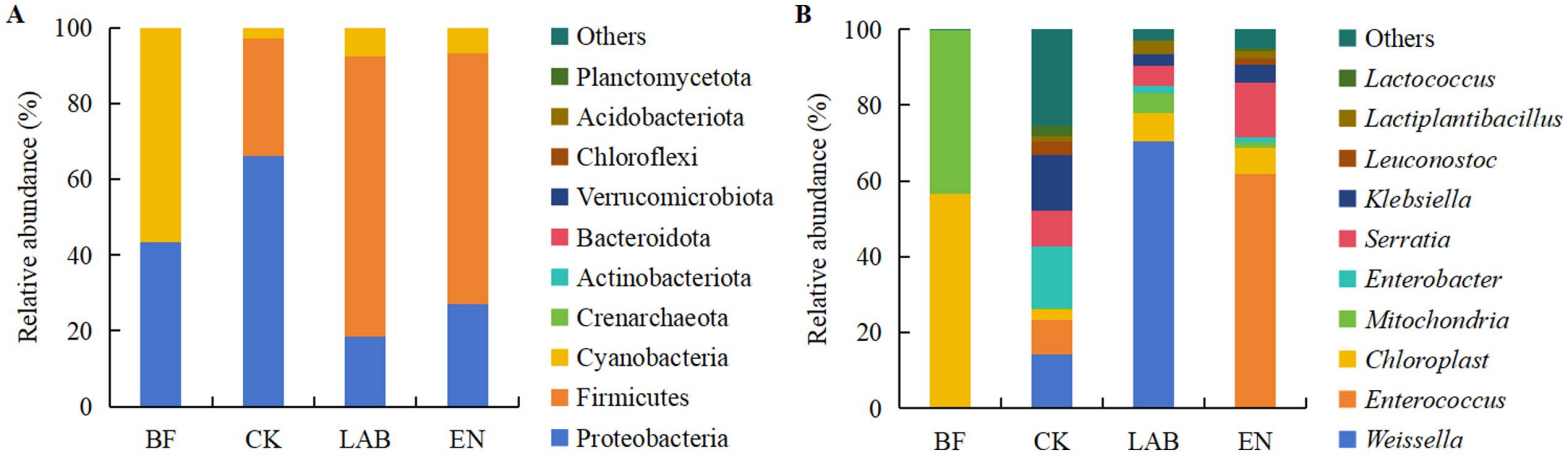
Relative abundance of bacterial composition in fresh and corn kernels after 60 days of wet storage at the **(A)** phylum and **(B)** genus level. BF, Fresh corn kernels; CK, The addition of deionized water; LAB, The addition of homo- and hetero-lactic acid bacteria; EN, The addition of cellulase.

The dominant phyla in fresh, high-moisture corn kernels were Cyanobacteria (56.5%) and Proteobacteria (43.4%) (Figure 3A; Supplementary Figure S1). In the CK treatment, the dominant phyla were Proteobacteria (66.1%) and Firmicutes (31.2%), while in the LAB and EN treatments, the dominant phyla were Firmicutes (70.1%) and Proteobacteria (22.8%). At the genus level, the dominant genera in fresh, high-moisture corn kernels were *Chloroplast* (56.5%) and *Mitochondria* (43.2%) (Figure 3B; Supplementary Figure S1). In the CK treatment, the most abundant genera were *Enterobacter* (16.3%) and *Weissella* (14.2%). In the LAB treatment, the abundant genus was *Weissella* (70.3%), while in the EN treatment, it was *Enterococcus* (61.7%).

**Figure 4:**
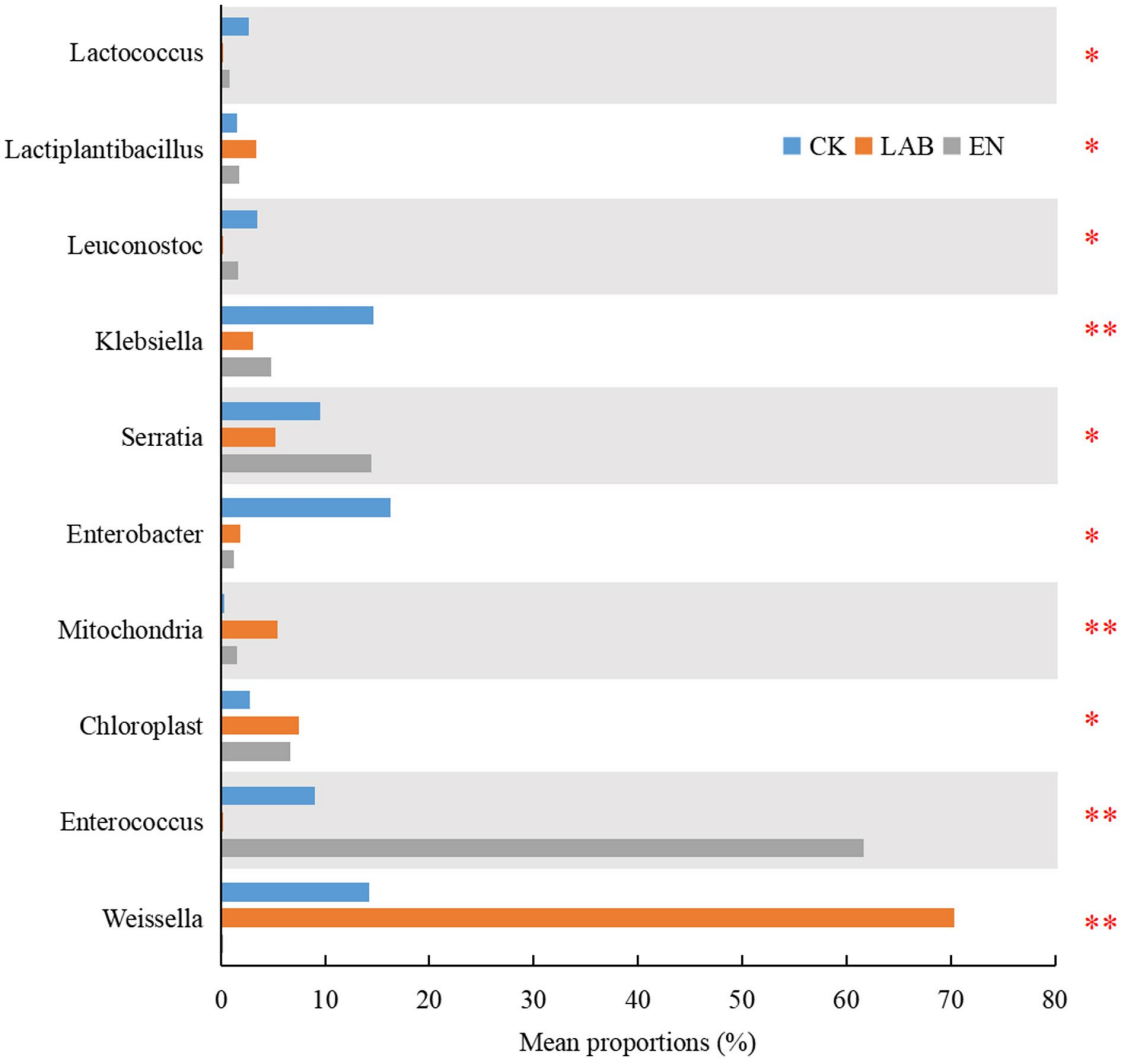
Comparison of different bacteria among the three treatments after 60 days of wet storage. CK, The addition of deionized water; LAB, The addition of homo- and hetero-lactic acid bacteria; EN, The addition of cellulase. *p < 0.05; **p < 0.01.

The proportion of *Weissella and Lactiplantibacillus* in the LAB treatment was higher than in the CK and EN treatments (Figure 4). In contrast, the proportion of *Enterococcus* was highest in EN, followed by CK, and then LAB treatment. The proportion of *Enterobacter* was higher in the CK treatment than in the LAB and EN treatments. Other bacteria (*Leuconostoc* and *Lactococcus*) showed significant differences between the CK and LAB treatments.

### Correlation analysis

**Figure 5:**
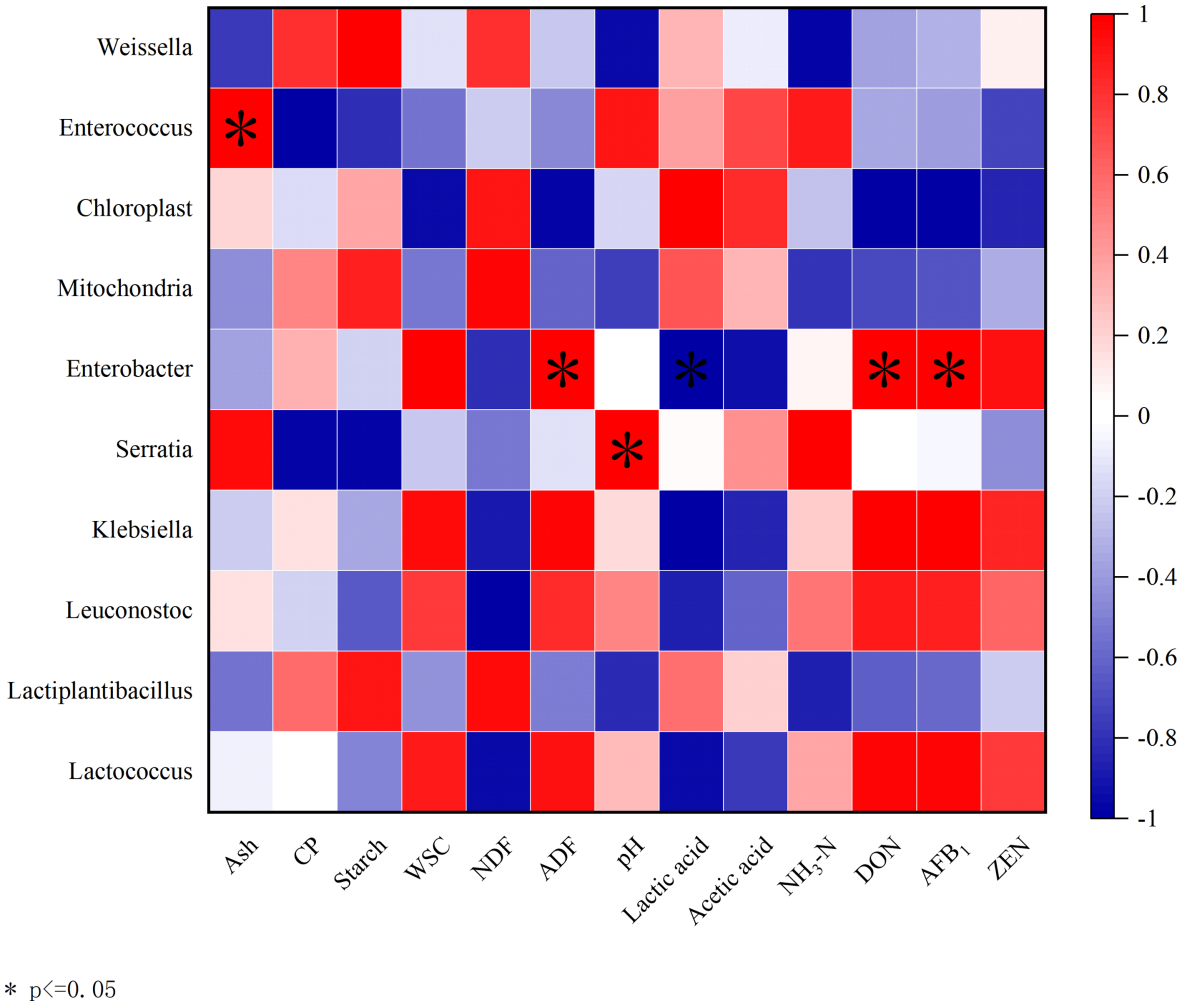
Correlation analysis between the bacterial community at genus level and chemical composition, fermentation characteristics, and mycotoxins. Ash, Crude ash; CP, Crude protein; WSC, Water-soluble carbohydrate; NDF, Neutral detergent fiber; and ADF, Acid detergent fiber. The corresponding value of the heatmap is the Spearman correlation coefficient *r* (−1 to 1). A value above 0 indicates a positive correlation (red), a value below 0 shows a negative correlation (blue), *p < 0.05.

A correlation analysis was conducted between the bacterial community at the genus level and chemical composition, fermentation characteristics, and mycotoxins to investigate the effects of the microbial community on fermentation quality (Figure 5). *Enterococcus* was positively correlated with ash (*R* = 1, *p* < 0.05). *Enterobacter* showed positive correlations with ADF (*R* = 1, *p* < 0.05), DON (*R* = 1, *p* < 0.05), and AFB_1_ (*R* = 1, *p* < 0.05), but a negative correlation with lactic acid (*R* = −1, *p* < 0.05). *Serratia* was positively correlated with pH (*R* = 1, *p* < 0.05).

## Discussion

To address global food security challenges and achieve sustainable development goals, agricultural production must enhance productivity while significantly reducing grain loss (Foley et al., 2011). However, most agricultural regions, particularly in developing countries with large populations (e.g., China), have historically prioritized crop productivity over reducing food loss (Godfray et al., 2010; Cui et al., 2018). With advancements in economics and technology in these areas, reducing food loss and waste has become increasingly recognized as a critical strategy for strengthening food security.

In this study, we analyzed the mycotoxin concentrations in high-moisture harvested corn kernels subjected to wet storage. Our findings revealed that both at harvest and after wet storage, mycotoxin concentrations in the corn kernels remained relatively low. Additionally, during wet storage, the inoculation of LAB enhanced the relative abundance of desirable *Weissella*, which improved quality by increasing lactic, acetic acid, CP, and starch contents while decreasing pH, NH_3_-N, WSC, and mycotoxins levels. This study provides important theoretical and technical support for better preserving high-moisture corn kernels.

The mycotoxin concentrations in high-moisture corn kernels were lower both before and after wet storage due to two key factors. First, before wet storage, high-moisture corn kernels were typically harvested with a grain moisture content of over 30%, which reduces the risk of fungal infection, growth, and toxin accumulation. This finding was consistent with that of prior research. Greater ear rot infection and higher levels of aflatoxins, DON, nivalenol, or fumonisins may be associated with delayed harvests for grain maize (Munkvold, 2003). Mycotoxin content tends to increase with delayed harvests, especially under rainy conditions.

Furthermore, fermentation is an effective process for reducing the mycotoxin content through enzymatic breakdown. DON levels can decrease significantly during fermentation by approximately 38–46% of the original content (Lancova et al., 2008). In some cases, well-preserved corn silage can lead to a significant 90% reduction in FB_1_ levels after 3 months of fermentation (Keller et al., 2013). Therefore, the inherently low levels of fungal toxins at harvest, combined with the capacity of fermentation to sustain these toxins reduced, create a cumulative effect that helps maintain consistently low toxin levels in corn kernels, regardless of the harvest or storage period. Moreover, in this study, the LAB treatment significantly decreased the concentrations of DON, AFB_1_, and ZEN compared with the CK treatment. This effect may be due to the efficacy of LAB as mycotoxin-degrading bacteria in silage. LAB have been used as silage additives to improve silage quality and act as organic mycotoxin binders (Weinberg et al., 2003). Weinberg et al. (2003) demonstrated that certain LAB strains in silage could even survive in rumen fluid. Many researchers have studied the potential of LAB in mycotoxin degradation. For example, Niderkorn et al. (2007) used eight *Lactobacilli* and three *Leuconostoc* to convert ZEA to *α*-zearalenol under conditions simulating corn silage *in vitro*, with *Lactiplantibacillus plantarum* achieving the highest conversion rate at 50%.

Compared to the CK treatment, the LAB treatment exhibited a significant decrease in pH value and NH_3_-N content, along with a notable increase in lactic and acetic acid levels. This indicated relatively superior wet storage effectiveness with LAB treatment (Bumbieris et al., 2017). The decline in pH in the LAB treatment was attributed to the inoculation of lactic acid bacteria. These lactic acid bacteria suppress the activity of harmful microorganisms, swiftly dominate the environment, and utilize glucose to produce lactic acid. This process accelerated lactic acid accumulation in wet storage, establishing an acidic environment early in storage (Jiang et al., 2020).

Moreover, NH_3_-N and organic acid contents reflected the transformation of proteins and carbohydrates in fermented fodder. The LAB treatment significantly shortened the growth duration of harmful *Clostridia*, thereby reducing their detrimental impact on starch and protein degradation and minimizing the breakdown of these components (Hashemzadeh-Cigari et al., 2014). Consequently, in this study, the LAB treatment exhibited significantly higher starch and CP contents compared to the other treatments.

Regarding chemical composition, the LAB treatment exhibited significantly lower level of ash than the other two treatments, lower level of ADF than the CK treatment, and notably lower levels than those in fresh, high-moisture corn kernels. In fermented maize, ash refers to the residual residue remaining after completely oxidizing all organic substances in samples at temperatures ranging from 550 to 600°C in a high-temperature furnace. This ash primarily comprises inorganic elements such as minerals, oxides, salts, and occasionally traces of sediments. The reduction in ash could be due to the metabolic byproducts generated by LAB or other microbes introduced during the LAB treatment, which may alter crude ash content. Alternatively, it could result from reactions between certain acid ions and specific inorganic salts, forming new salts with reduced molecular weights, thus reducing crude ash content (Zhang et al., 2023).

Additionally, the decrease in ADF content in the wet-stored maize is likely due to the hydrolytic action of organic acids during fermentation, which reduces the digestible cell wall fractions. The acid hydrolysis of structural carbohydrates is typically accompanied by the release of WSC (Desta et al., 2016). However, in this study, the WSC content in the LAB and EN treatments was lower than in the CK group. This was primarily attributed to the dynamic changes in WSC content during fermentation. WSC was released through acid hydrolysis of fiber fractions and utilized by lactic acid bacteria for organic acid production (Jiang et al., 2020).

Nevertheless, the WSC content in the LAB treatment was significantly lower than in the CK treatment and notably lower than in fresh, high-moisture corn kernels. This reduction was predominantly due to the rapid fermentation of WSC into lactic acid by *Lactiplantibacillus plantarum* during the LAB treatment. Subsequently, in later stages, *Weissella* took dominance, engaging in heterofermentation and further depleting substrates. This outcome was consistent with the findings of Blajman et al. (2018).

In our study, we observed a decrease in bacterial diversity (Observed features and Chao1) in the LAB and EN treatment after 60 days compared to the CK treatment. As expected, the unique and common bacterial OTUs showed the same variation trend, consistent with previous research (Xu et al., 2019). In their study, corn silage indicated a reduction in bacterial diversity. The bacterial diversity decreases after successful fermentation, possibly due to the LAB treatment, which improves the fermentation of silages (Guan et al., 2018). The additives in LAB treatment affected silage quality by changing the bacterial profile. In this study, the LAB treatment resulted in higher lactic acid and acetic acid contents and a lower pH value compared to the CK treatment, suggesting a mixed fermentation pattern, both homo- and heterofermentative. The low pH and high lactic acid content in the fermentation of corn with the addition of *Lactiplantibacillus plantarum* and *Weissella* likely stemmed from the rapid fermentation by lactic acid bacteria, which converted WSC into lactic acid, rapidly reducing the pH. In the later fermentation stages, the heterofermentative bacteria *Weissella* became dominant, shifting the fermentation from lactic acid to acetic acid production. The higher level of *Weissella* in the LAB (70.3%) treatment after 60 days confirmed these results.

In our study, an intriguing finding emerged. Despite lower levels of lactic acid and acetic acid in the EN treatment, the pH remained higher. This observation might be explained by the composition of the microbial community. After 60 days of fermentation, *Enterococcus* dominated the EN treatment (61.7%). Prior research has suggested that EN supplementation could partially facilitate cellulose breakdown, generating simple sugars and oligosaccharides that may provide additional nutrients favorable to *Enterococcus* growth. These carbon sources might favor specific strains, potentially leading to their proliferation. *Enterococcus* is capable of protein degradation, which results in NH_3_-N production. In this study, the NH_3_-N in the EN treatment was significantly higher than in the other two treatments, while the CP content was lower. This result contrasts with previous research. Romaní et al. (2006) indicated that adding cellulase during ensiling not only provides ample sugar sources for fermentation, promoting lactic acid fermentation, but also reduces the content of ADF and hemicellulose in silage, improving feed digestibility, and subsequently enhancing livestock productivity. Several factors, such as cellulase concentration, cellulase activity, fermentation temperature, and fermentation substance, might contribute to this disparity (He et al., 2018; He et al., 2019).

## Conclusion

We systematically investigated variations in fermentation quality, mycotoxin concentrations, and microbial communities during the wet storage of high-moisture corn kernels under different additive treatments. Among the treatments, LAB addition significantly improved the fermentation quality of high-moisture corn kernels. The inoculation of LAB enhanced the relative abundance of the desirable *Weissella* genus, which contributed to increased levels of lactic acid, acetic acid, CP, and starch while reducing pH, NH_3_-N, WSC, and mycotoxins concentrations. Overall, LAB addition can effectively preserve nutrients during wet storage and improve fermentation quality, which provides valuable theoretical support and practical guidance for future applications in the wet storage of high-moisture corn kernels.

## Data Availability

The original contributions presented in the study are publicly available. This data can be found at: https://www.ncbi.nlm.nih.gov/bioproject/PRJNA1186269.
