# Supplementary figures and images for: Effects of different additives on fermentation quality, mycotoxin concentrations, and microbial communities in high-moisture corn kernels during wet storage
Source: Front Microbiol. 2024 Dec 4;15:1508842. doi: 10.3389/fmicb.2024.1508842 (PMC11652497; doi:10.3389/fmicb.2024.1508842)

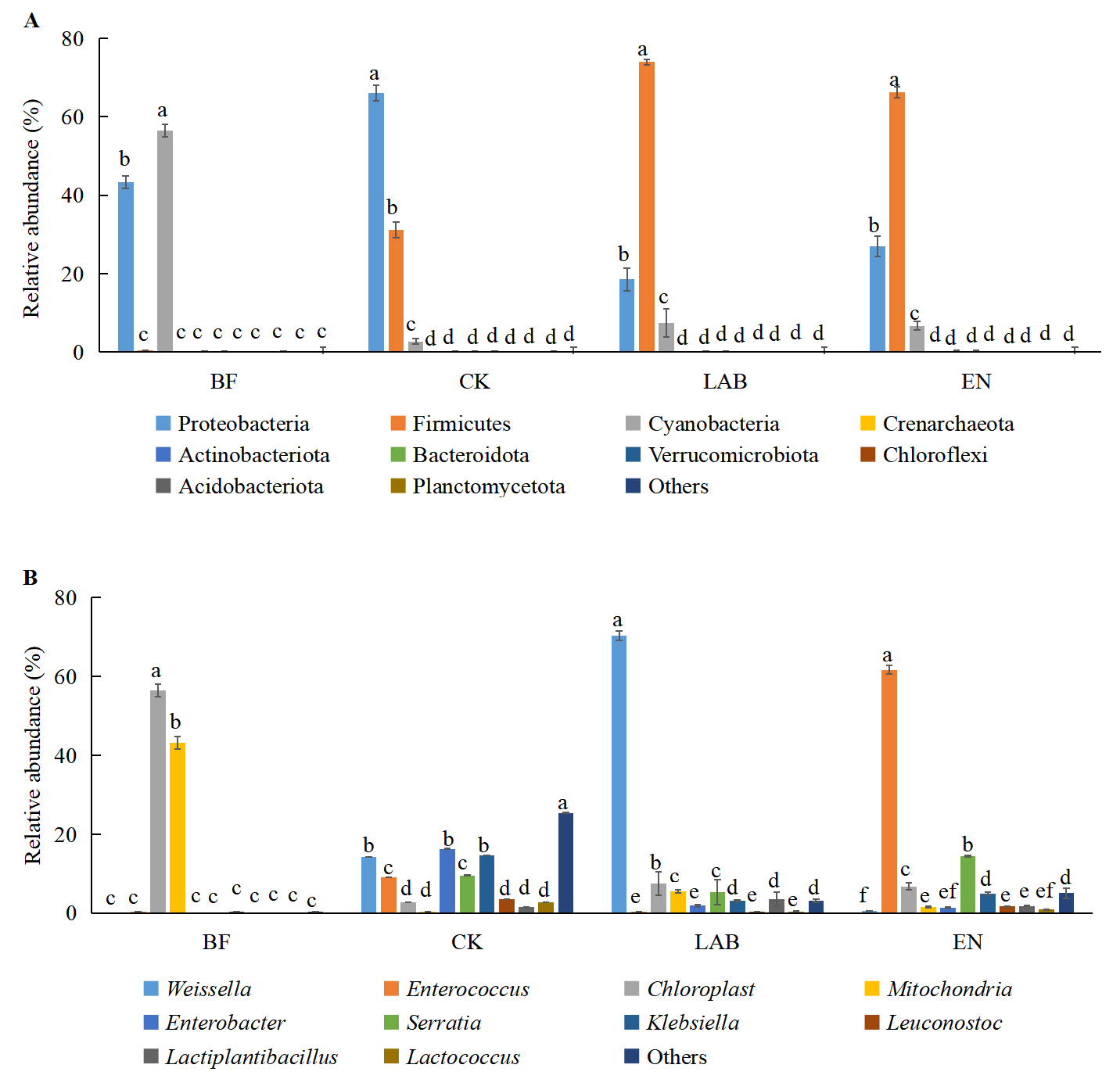

Supplement: Supplementary file 1 [file Image_1.TIF]

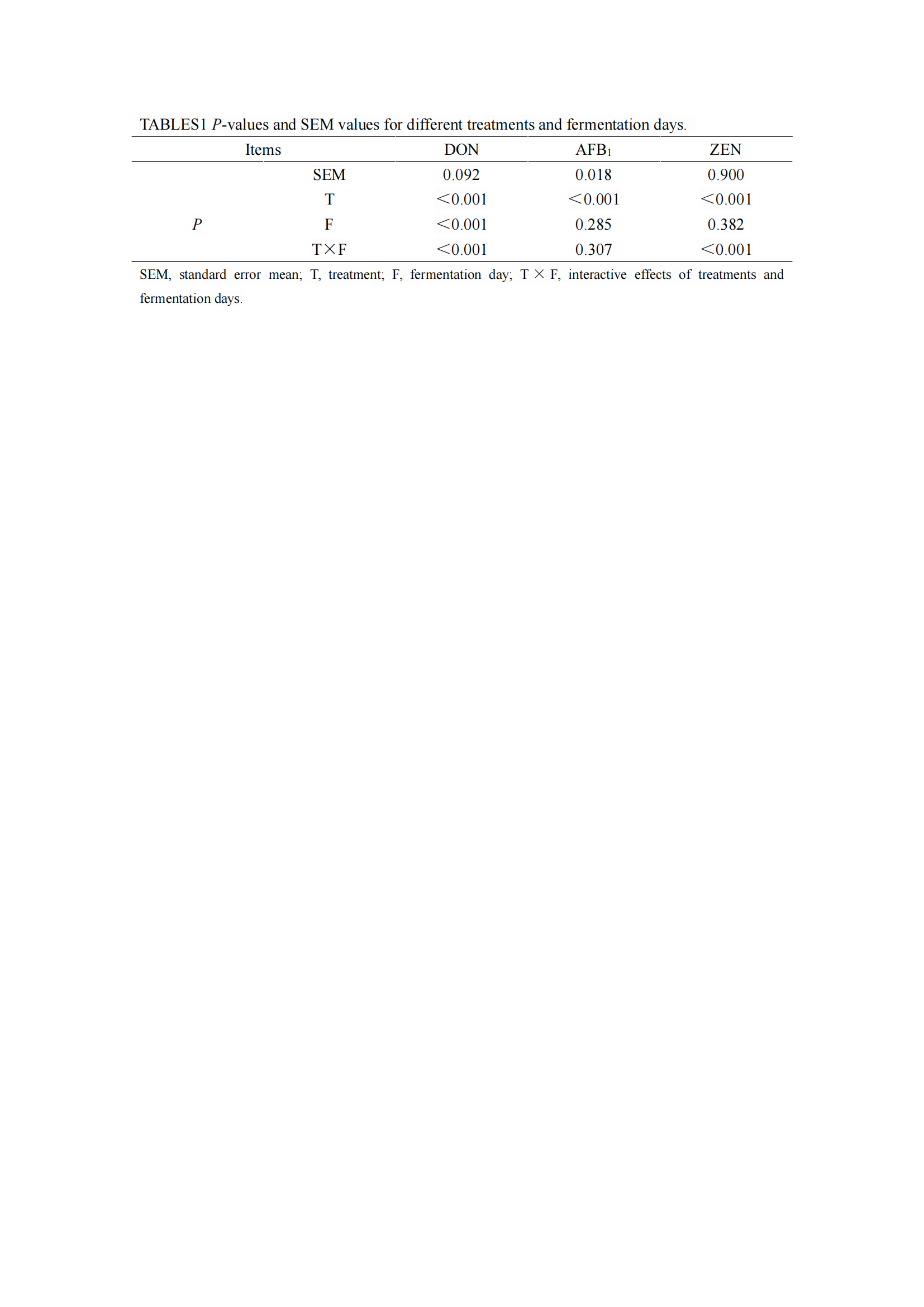

Supplement: Supplementary file 2 [file Image_2.TIF]
